# Good? A very long COVID-19

**DOI:** 10.70962/jhi.20250178

**Published:** 2025-12-26

**Authors:** Quentin Philippot, Marie-Pierre Debray, Alice Guyard, Antoine Achkar, Tom Le Voyer, Quentin Le Hingrat, Bruno Crestani, Raphaël Borie

**Affiliations:** 1 Service de Pneumologie, Allergologie et Transplantation, Hôpital Bichat Claude Bernard, AP-HP, Paris, France; 2 Université Paris Cité, Inserm, UMR 1149, Centre de Recherche de l’Inflammation, Paris, France; 3 Service de Radiologie, Hôpital Bichat Claude Bernard, AP-HP, Paris, France; 4 Département de Pathologie, Hôpital Bichat Claude Bernard, AP-HP, Paris, France; 5 Service de Pneumologie et Oncologie Thoracique, Centre Hospitalier Intercommunal Poissy Saint Germain, Poissy, France; 6 St. Giles Laboratory of Human Genetics of Infectious Diseases, The Rockefeller University, New York, New York, USA, Laboratory of Human Genetics of Infectious Diseases, Inserm U1163, Paris, France; 7 Service de Virologie, Hôpital Bichat Claude Bernard, AP-HP, Paris, France; 8 Université Paris Cité, Inserm, IAME, Paris, France

## Abstract

We report a case of chronic SARS-CoV-2 infection resulting from impaired B cell–mediated immunity to SARS-CoV-2 in a patient with Good syndrome. Immunoglobulin replacement therapy alone was sufficient to achieve clinical and radiological resolution.

## Teaser

We report a case of chronic pulmonary SARS-CoV2 infection caused by impaired B cell–mediated immunity to SARS-CoV-2 in a patient with Good syndrome. Immunoglobulin replacement therapy alone was sufficient to achieve clinical and radiological resolution.

## Case presentation

**Figure 1.**
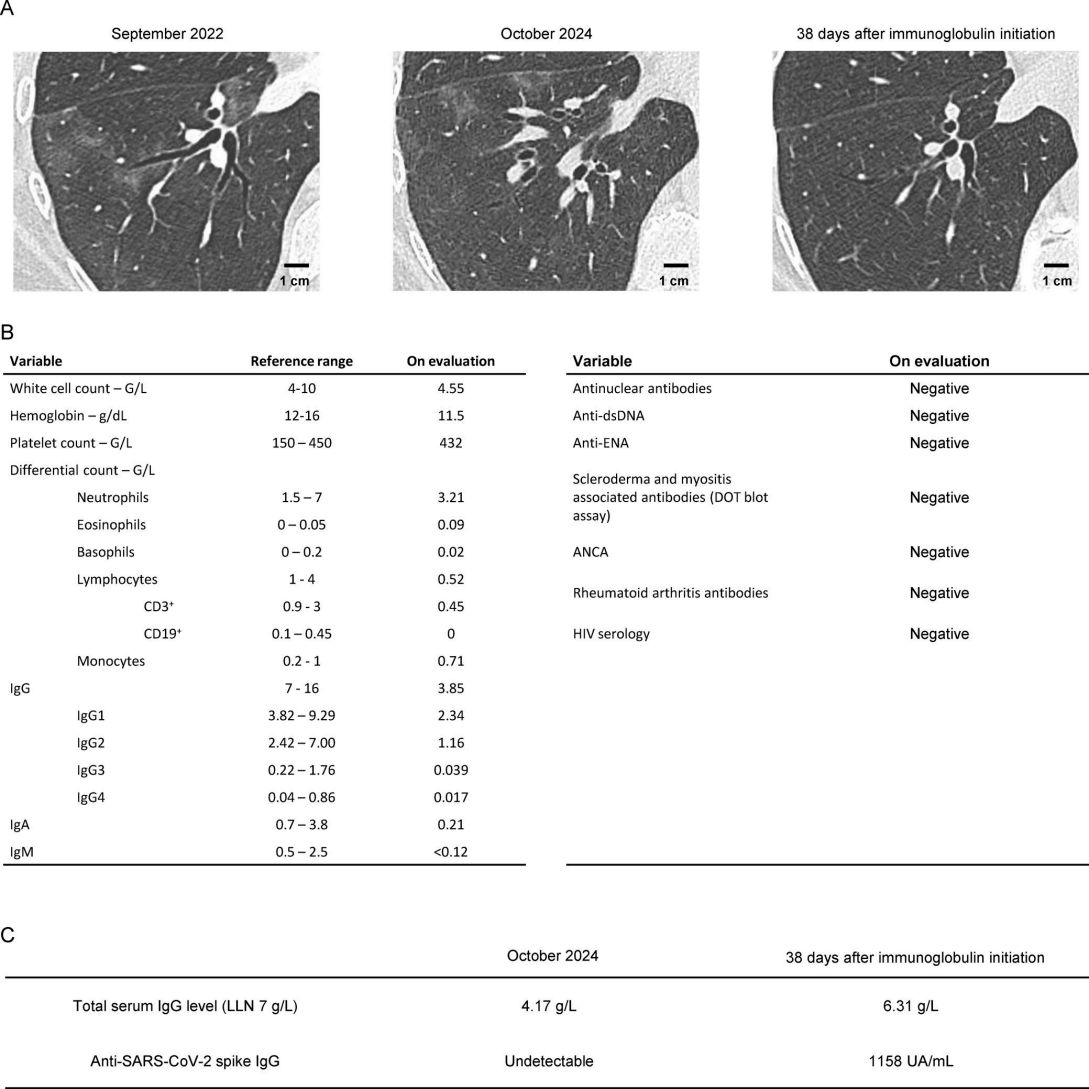
**Chest CT scan and laboratory data from the patient. (A)** Chest CT scanners of the patient at the start of her respiratory symptoms (left), referral to our center (middle), and 38 days after immunoglobulin replacement therapy initiation (right). **(B)** Laboratory data. These data were collected in 2024. The patient’s initial blood test for immunoglobulin concentration, performed in December 2023, showed a level of 3.4 g/L. LLN, lower limit of normal. **(C)** Total serum IgG level and anti-SARS-CoV2 spike IgG level in the patient's serum before and after immunoglobulin replacement therapy initiation. dsDNA, double-stranded DNA; ENA, extractable nuclear antigens; ANCA, anti-neutrophil cytoplasmic antibodies.

A 57-year-old woman was referred to our center for an interstitial lung disease (ILD) diagnosis in 2024. She only reported a history of irritable bowel disease. She was a former smoker. No other pneumotoxic exposure was found in her environment. Since 2021, she presented recurrent cystic infections and vaginal herpetic recurrencies. In March 2022, she presented asthenia, upper respiratory track symptoms, and cough. A SARS-CoV2 PCR from a nasal swab was positive, confirming a diagnosis of mild COVID-19. Her symptoms resolved spontaneously within a few weeks. In June 2022, the patient experienced a second episode of mild COVID-19 with similar symptoms. Despite the SARS-CoV2 PCR from the nasal swab becoming negative, the patient continued to experience persistent cough and dyspnea, which prompted further evaluation and revealed ILD along with a thymic compartment mass on computed tomography (CT) imaging (Fig. 1 A). Thymectomy was performed, and a diagnosis of AB thymoma (Masaoka-Koga stage IIa, American Joint Commission on Cancer stage pT1aNxR0) was made with complete resection. Due to the persistence of the ILD, the patient was evaluated at our hospital in October 2024. She described cough associated with dyspnea. Her treatment did not include any drugs associated with lung toxicity. No case of ILD was reported in her family. The transthoracic cardiac ultrasound was normal. The laboratory results revealed a lymphopenia with undetectable CD19^+^ and decreased CD3^+^ lymphocytes associated with a hypogammaglobulinemia (IgG 3.85 g/L, IgA 0.21 g/L, and IgM < 0.12 g/L), never tested before. HIV serological test was negative. There was no serologic argument to suggest a connective tissue disease (Fig. 1 B). The cytological analysis of the bronchoalveolar lavage fluid (BAL) showed lymphocytic alveolitis (800,000 cells per milliliter, including 43% of macrophages, 52% of lymphocytes [3% of CD3^+^CD4^+^, 90% of CD3^+^CD8^+^, 0% of CD19^+^, and 6% of CD3^−^CD16^+^CD56^+^], 2% of neutrophils, and 1% of eosinophils). Microbiological analyses of the BAL fluid were positive for SARS-CoV2 and negative for all other pathogens tested (including bacteria, mycobacteria, herpes viruses, and *Pneumocystis jirovecii*). After multidisciplinary team discussion, a diagnosis of chronic pulmonary SARS-CoV2 infection associated to Good syndrome was raised.

To support our diagnosis, we performed next-generation sequencing of the SARS-CoV2 genome detected in the patient’s BAL fluid, and the strain was identified as a 21L variant (Omicron BA.2; https://clades.nextstrain.org). This variant was circulating in France from January to August 2022. This overlaps with the period when the patient’s respiratory symptoms began, which subsequently persisted for approximately two years. Circulating autoantibodies against type I IFNs may underlie life-threatening COVID-19 pneumonia and have been observed in patients affected of thymoma (1, 2). No autoantibodies neutralizing IFN-α2, IFN-ω, or IFN-β were detected in the patient’s plasma obtained in 2024. This observation further highlights that immunological mechanisms protecting against life-threatening COVID-19 pneumonia differ from those involved in controlling chronic pulmonary SARS-CoV2 infection. From this, we hypothesized that the chronic infection was attributable, at least in part, to hypogammaglobulinemia and B cell lymphopenia, although a contributory role of T cell lymphopenia could not be excluded. Immunoglobulin replacement therapy was therefore initiated. Following treatment, SARS-CoV2 spike–specific IgG, which was previously undetectable, was detected in the patient’s serum. Her respiratory symptoms improved, paralleled by pulmonary function test improvement, with a mild increase in forced vital capacity (3.85 L to 3.93 L) and diffusing capacity for carbon monoxide (61% to 68%). The ILD resolved, though we did not confirm the negativation of SARS-CoV2 PCR in BAL fluid for ethical reasons (Fig. 1, A and C).

## Case discussion

Good syndrome, the association between a thymoma and an immunodeficiency, was first described by Robert Good in 1954, who reported a case of thymoma and hypogammaglobulinemia in an adult. Like common variable immunodeficiency (CVID), Good syndrome is characterized by hypogammaglobulinemia. In addition to being prone to pulmonary infections, patients with CVID may also develop ILD, particularly granulomatous-lymphocytic ILD (GLILD), the prevalence of which varies depending on the genotype. Unlike CVID patients, low, or absent, B cells in the peripheral blood are almost constant in Good syndrome patients, and defects in T cell–mediated immunity are also reported (3). In addition, lymphoid hyperplasia seems infrequent and GLILD extremely rare in Good syndrome patients (3).

ILD has been reported in patients with thymoma, likely due to disrupted central immune tolerance. Thymoma-associated ILD was therefore first considered in this case, but this diagnosis was revised after SARS-CoV2 was detected in the BAL fluid. Chronic pulmonary SARS-CoV2 infection has never been reported in a patient with Good syndrome but has been previously reported in patients with primary or secondary B cell–mediated immunity deficiency. These clinical observations suggest that B cell–mediated immunity plays a crucial role in controlling chronic SARS-CoV2 replication in the lungs, whereas type I IFN–mediated immunity is essential for protection against life-threatening COVID-19 (1, 2, 4, 5). Supporting this, our patient did not develop severe COVID-19 pneumonia or any other form of severe viral pneumonia. Her respiratory condition improved following immunoglobulin replacement therapy, likely due to the passive transfer of SARS-CoV2–specific IgG.

### Conclusion

We identified a case of very long COVID-19 caused by impaired B cell–mediated immunity to SARS-CoV2 in a patient with Good syndrome. Immunoglobulin replacement therapy alone was sufficient to achieve clinical and radiological resolution.

## Data Availability

No new data were generated or analyzed in support of this study.

## References

[1] Bastard, P., L.B. Rosen, Q. Zhang, E. Michailidis, H.H. Hoffmann, Y. Zhang, K. Dorgham, Q. Philippot, J. Rosain, V. Béziat, . 2020. Autoantibodies against type I IFNs in patients with life-threatening COVID-19. Science. 370:eabd4585. 10.1126/science.abd4585 32972996 PMC7857397

[2] Bastard, P., A. Gervais, T. Le Voyer, Q. Philippot, A. Cobat, J. Rosain, E. Jouanguy, L. Abel, S.-Y. Zhang, Q. Zhang, . 2024. Human autoantibodies neutralizing type I IFNs: From 1981 to 2023. Immunol. Rev. 322:98–112. 10.1111/imr.13304 38193358 PMC10950543

[3] Malphettes, M., L. Gérard, L. Galicier, D. Boutboul, B. Asli, R. Szalat, A. Perlat, A. Masseau, N. Schleinitz, G. Le Guenno, . 2015. Good syndrome: an adult-onset immunodeficiency remarkable for its high incidence of invasive infections and autoimmune complications. Clin. Infect. Dis. 61:e13–e19. 10.1093/cid/civ269 25828999

[4] Brodin, P., G. Casari, L. Townsend, C. O’Farrelly, I. Tancevski, J. Löffler-Ragg, T.H. Mogensen, J.L. Casanova, and COVID Human Genetic Effort. 2022. Studying severe long COVID to understand post-infectious disorders beyond COVID-19. Nat. Med. 28:879–882. 10.1038/s41591-022-01766-7 35383311

[5] Zhang, Q., P. Bastard, Z. Liu, J. Le Pen, M. Moncada-Velez, J. Chen, M. Ogishi, I.K.D. Sabli, S. Hodeib, C. Korol, . 2020. Inborn errors of type I IFN immunity in patients with life-threatening COVID-19. Science. 370:eabd4570. 10.1126/science.abd4570 32972995 PMC7857407

